# A case of secondary syphilis misdiagnosed as psoriasis vulgaris: Discover the real cause beyond the surface with reflection confocal microscopy and leave the great imitator nowhere to hide

**DOI:** 10.1111/srt.13374

**Published:** 2023-05-28

**Authors:** Jianfeng Zhang, Xia Li, Dongmei Shi

**Affiliations:** ^1^ Shandong First Medical University & Shandong Academy Of Medical Sciences Jinan China; ^2^ Sishui Dermatology Prevention Station Sishui China; ^3^ The Laboratory of Medical Mycology Jining No.1 People's Hospital Jinig China; ^4^ Department of Dermatology Jining No.1 People's Hospital Jining China

Dear Editor,

Syphilis is a chronic infectious disease caused by *Treponema pallidum* that can affect multiple systems and have a variety of clinical manifestations. Secondary syphilis is caused by *Treponema pallidum* blood and lymph transmission, which occurs after several weeks or months of contact (3–10 weeks). The most common clinical manifestation (80%) is a generalized non‐pruritic scaly papule, ranging in size from 1–2 to 15–20 mm and color from pink to purplish red to brownish red. Secondary syphilis skin manifestations vary but commonly include macules, maculopapular rash, papules and plaques.^1^ Lesions can be generalized or confined, and they can mimic the lesions of many diseases, such as pityriasis rosea,^2^ verruca vulgaris,^3^ annular lichen planus,^4^ which are referred to as the Great Imitator or Great Mimicker.^5^ A syphilis diagnosis is often based on a suggestive clinical history and supportive laboratory^6^ (that is, serodiagnosis) tests, especially, secondary syphilis often causes rash on palms and soles. Unfortunately, it is used as a differential diagnosis by some doctors and lead to the occurrence of misdiagnosis.

Reflection confocal microscopy (RCM for short) is a three‐dimensional computed tomography technique based on the principle of optical confocal imaging, that is widely used for the diagnosis of skin tumors and various inflammatory skin diseases such as contact dermatitis, psoriasis, and discoid lupus erythematosus.^7^ RCM is accomplished by emitting a low‐powered infrared laser (830 nm) to achieve a lateral resolution of approximately 1 μm, resulting in images that are nearly 30 times magnified than high magnification microscopy images up to 150–350 μm in‐depth, displaying black and white images due to the tissue's different refraction and reflection coefficients to the laser.^8^ As far as we know, an RCM evaluation of secondary syphilis without rash on palms or soles has not yet been reported.

The patient of our case was a 15‐year‐old male student. Six months ago, after an unclean sexual encounter, the patient developed red papules on the penis and experienced pruritus, which he ignored because it went away on its own without any intervention. Two months ago, the patient developed purplish red papules on his trunk (Figure 1A), limbs, and external genitalia (Figure 2A), along with a small number of scales and slight conscious pruritus but no other special discomforts. There was no rash on palms or soles. He was diagnosed with psoriasis vulgaris in a local hospital and treated with oral anti‐silver capsules and topical glucocorticoid cream for one month, but without success. Then he come to our outpatient clinic and underwent reflection confocal microscopy. A slender and bright rod&W‐shaped structure with regular alternation was found in the epidermis (Figure 2B), meanwhile we took a pathological examination of his abdominal lesions and the pathology of abdominal tissue showing: mild hyperkeratosis of epidermis, hypertrophic acanthosis, dermal vascular dilatation, more lymphocytes infiltration around, and occasional plasma cellindicating spirochete infection (Figure 3), indicating spirochete infection, so we recommended the patient to have a serological examination of syphilis. Then he had a test for syphilis: TRUST 1:64 and TPPA +. Without any doubt, the patient was diagnosed with secondary syphilis, and was treated with benzathine penicillin for three weeks before the lesions faded, resulting in pigmentation (Figures 1B and 2C). In the meantime, RCM examination at the same site showed that the thin‐and‐long bright rod&W‐shaped structures and inflammatory cell infiltration in the epidermis had disappeared (Figure 2D).

**FIGURE 1:**
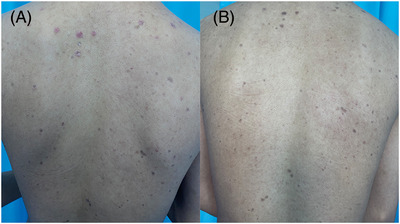
Comparison of patients with back rash before (A) and after treatment (B).

**FIGURE 2:**
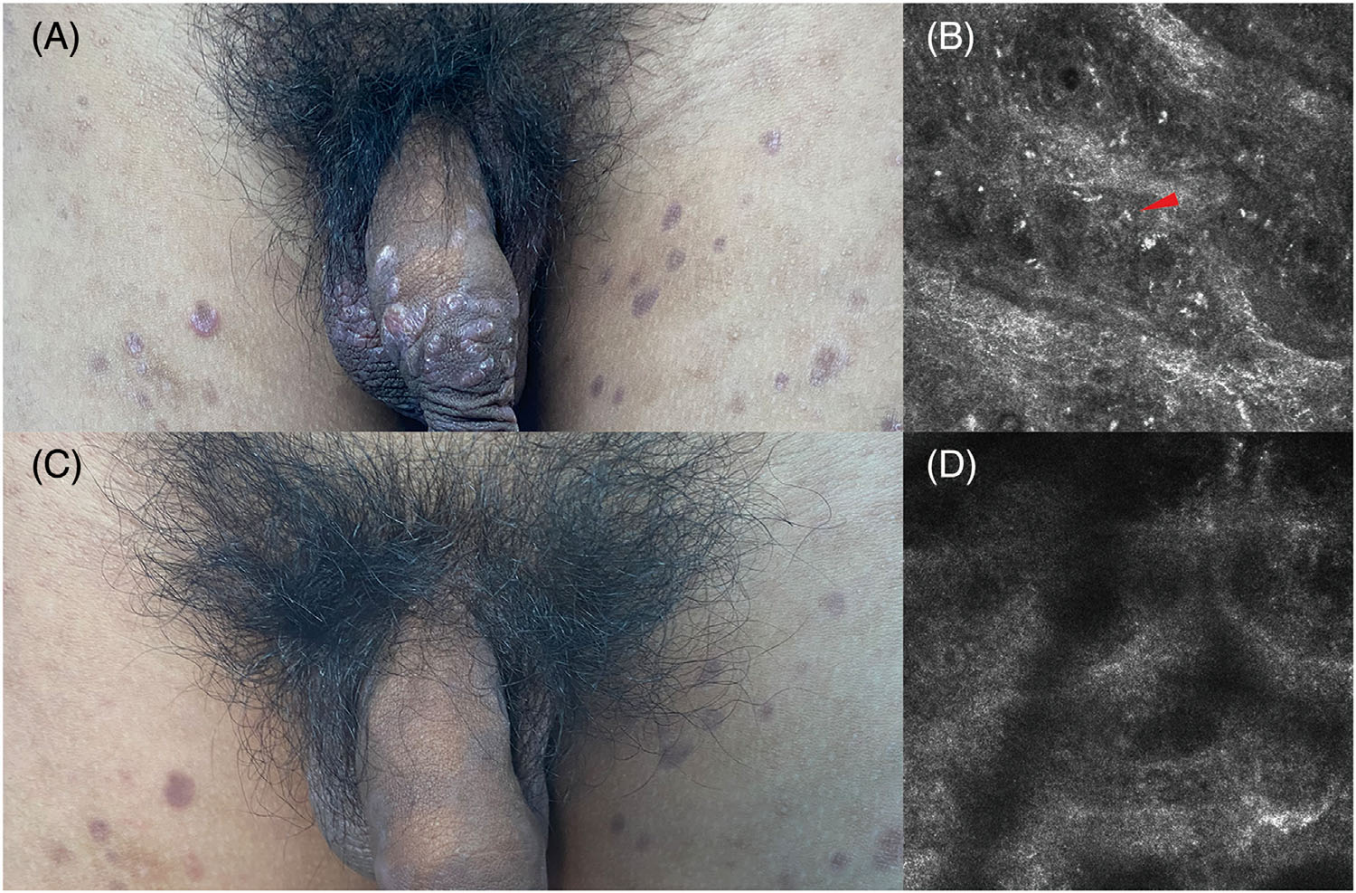
Comparison of patients with scrotal rash before (A) and after treatment (C).

**FIGURE 3:**
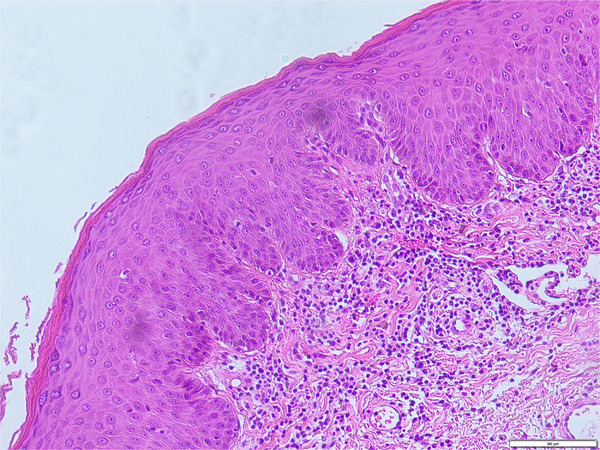
Mild hyperkeratosis of epidermis, hypertrophic acanthosis, dermal vascular dilatation, more lymphocytes infiltration around, and occasional plasma cellindicating spirochete infection (HE, 20×).

Through RCM imagery, we can visually observe *Treponema pallidum* in the epidermis and *Treponema pallidum* presents as slender and bright rod&W‐shaped structure (Figure 2B). Due to the helical structure of *Treponema pallidum* and the characteristics of RCM horizontal tomography, *Treponema pallidum* shows a W‐shape (Figure 2B). Besides, *Treponema pallidum* has high refraction due to its protein‐rich outer mold; it is highlighted in the RCM images and easy to distinguish. We also randomly measured 20 lengths of these structures in the RCM images of the patient; they ranged from 7–10um, which was consistent with the length range of *Treponema pallidum*. In conclusion, RCM is an excellent predictor of atypical secondary syphilis and can help to avoid misdiagnosis and missed diagnosis.

## CONFLICT OF INTEREST STATEMENT

The authors declare that there is no conflict of interest that could be perceived as prejudicing the impartiality of the research reported.

## FUNDING INFORMATION

The authors received no specific funding for this work.

## ETHICS STATEMENT

The patient signed an informed consent form. This article has been approved by the institutional review board.

## Data Availability

Data sharing is not applicable to this article as no new data were created or analyzed in this study.
